# Influence of pH-Responsive Monomer Content on the Behavior of Di-Block Copolymers in Solution and as Stabilizers of Pickering Latex Particle Emulsifiers

**DOI:** 10.3389/fchem.2018.00301

**Published:** 2018-07-20

**Authors:** Mohamed S. Manga, Olivier J. Cayre, Simon Biggs, Timothy N. Hunter

**Affiliations:** ^1^Faculty of Engineering, School of Chemical and Process Engineering, University of Leeds, Leeds, United Kingdom; ^2^The University of Western Australia, Perth, WA, Australia

**Keywords:** pH-responsive polymer, pDMAEMA, core-shell particles, Pickering emulsions, membrane emulsification

## Abstract

In this study, diblock copolymers poly(methyl methacrylate)—block—poly (2-dimethylaminoethyl methacrylate) (pMMA-b-pDMAEMA) are investigated for the steric stabilization of latex particles and the subsequent use of these latex particles as Pickering emulsifiers. Solution properties of the diblock copolymers highlight that the pDMAEMA block length influences the critical micelle concentration (CMC) and micelle hydrodynamic diameter in response to changes in pH and the p*K*_a_. The block length can also be used as a way to control the particle size of sterically stabilized polystyrene latex particles prepared via emulsion polymerization. The suspension properties of these latex particles are also presented. Emulsion studies using these latex particles as emulsifiers show that both continuous phase pH and electrolyte concentration affect emulsion stability to coalescence. At high pH, stable emulsions are formed due to the affinity of the particles to the interface. At low pH, protonation of the amine groups reduces the affinity and thus droplet coalescence is observed. Increasing the electrolyte concentration improves emulsion stability, but causes an increase in droplet size due to adsorption of flocculated/aggregated particles. Finally, it is shown that these latex particles can be used in conjunction with membrane emulsification techniques to produce emulsions with low polydispersity.

## Introduction

The preparation and stabilization of emulsions and foams using colloidal particles (commonly referred to as Pickering systems) has been well documented for over a century (Ramsden, 1903; Pickering, 1907; Binks and Horozov, 2006; Hunter et al., 2008; Dickinson, 2010; Lam et al., 2014; Binks, 2017). For interfacial stabilization to occur, these Pickering stabilizers self-assemble at the fluid-fluid interface and remain irreversibly adsorbed once attached, preventing coalescence (Finkle et al., 1923; Binks and Horozov, 2006). In addition, their size and wettability (defined by their contact angle) play an important role in dictating the amount of energy that is required to detach them from the interface (Binks, 2002; Binks and Horozov, 2006).

The majority of research into Pickering systems has been conducted with inorganic particles such as silica, owing to their well-defined shape, availability of different sizes and ability to chemically tune the surface properties (Binks and Lumsdon, 1999; Binks and Horozov, 2005). The use of organic particles (e.g., polymer latex) as stabilizers has also received significant attention (Velev et al., 1996; Velev and Nagayama, 1997; Dinsmore et al., 2002), and as a consequence of developments in polymer chemistry, this has further expanded to include stimuli-responsive latex systems (Tang et al., 2015; Fujii and Nakamura, 2017). A key driver for this development, is the ability to form new “smart” materials with these responsive particles such as microcapsules (Biggs et al., 2008; San Miguel et al., 2010; Thompson et al., 2010, 2015; Cayre et al., 2012).

While there are numerous examples of Pickering emulsions produced using stimuli-responsive latex emulsifiers in the literature with various stimuli, the focus on this present study is on pH-responsive systems. Tu and co-workers developed pH-responsive Janus polymeric particles based on polystyrene and polyacrylic acid (Tu and Lee, 2014). They report that the particles can change shape based on changes in suspension pH due to the protonation/deprotonation of the acrylic acid. When used as emulsifiers, it was demonstrated that these particles were able to induce phase inversion of the emulsions when the continuous phase pH was switched. The authors stated that this occurred due to protonation of the acrylic acid at low pH (lowering charge) increasing wettability with oil phase to form a w/o emulsion, whilst deprotonation occurred at high pH (increasing charge) leading to o/w emulsions. Microgel latex particles have been successfully utilized by numerous groups with the most extensively studied being poly(N-isopropylacrylamide) based microgels (Li and Ngai, 2011; Liu et al., 2012; Richtering, 2012). Other similar systems that have been studied are poly(4-vinylpyridine)/silica (P4VP/SiO_2_) (Fujii et al., 2005, 2006), and 2-vinylpyridine (2VP) (Dupin et al., 2007), where emulsion stability is based on the continuous phase pH used in relation to the p*K*_a_ of the polymer. For example in the case of P4VP/SiO_2_, lowering the pH below the p*K*_a_ caused the particles to protonate and desorb from the interface leading to emulsion instability. A similar mechanism exists with cross-linked poly(tert-butylamino)ethyl methacrylate (pTBAEMA) latex emulsifiers (Morse et al., 2012).

Alternatively, poly(methyl methacrylate)—block—poly(2-dimethylaminoethyl methacrylate) (pMMA-b-pDMAEMA) has previously been used as a pH-responsive steric stabilizer for the synthesis of polymer latex particles by Armes et al. and the current authors (Amalvy et al., 2003, 2004; Read et al., 2004; Reis et al., 2010; Cayre et al., 2012). The pDMAEMA block is pH-responsive exhibiting a p*K*_a_ of 7–7.5 (Baines et al., 1996), which influences its behavior in solution and when used as a steric stabilizer. As a result, these latex particles have been used as emulsifiers to produce pH-responsive emulsions (Amalvy et al., 2003; Cayre et al., 2012), i.e., a similar mechanism to that of P4VP and pTBAEMA described above.

To date, there are very few studies that have investigated the influence of polymer chain length on the adsorption behavior of sterically stabilized particles at fluid-fluid interfaces. Reed et al. (2012) observed the influence of latex particles sterically stabilized with different chain lengths of a non-ionic macromonomer, pGMA (glycerol monomethacrylate) on particle wettability at fluid-fluid interfaces. Here, the interfacial contact angle was measured using a gel trapping technique (GTT) and a film caliper method (FCM). The equilibrium interfacial particle contact angle was found to be insensitive to pGMA chain length, because the high grafting densities achieved during particle synthesis meant that the polymer brushes were compact, forming a dense surface layer. The dense layer limited the access of the non-polar solvent to the particle surface and hence changes in the interfacial contact angle was found to be limited. Alternatively, Saigal et al. (2010), investigated inorganic silica particles stabilized with a pDMAEMA homopolymer as a thermo-responsive Pickering emulsifier. At high pH values, pDMAEMA has a low critical solubility temperature above which particle dispersions begin to flocculate. It was found that the pDMAEMA content at similar grafting densities did not affect emulsion stability regardless of the oil-type or emulsification temperature used at a continuous phase pH of 7–9.

It is evident that while the role of responsive polymer chain length may be critical to defining latex particle behavior and overall Pickering emulsion stability, grafted chain interactions are complex and difficult to predict from the free polymer behavior. To further understand these links, we present a comprehensive study that tracks the effect of a pH-responsive block on polymer solution behavior to bulk resulting effects on latex particles as stabilizers and finally to their performance as Pickering emulsifiers. While previous studies have investigated the use of well-defined steric stabilizers to exert control over the synthesis of sterically-stabilized latexes and covalently cross-linkable colloidosomes (Thompson and Armes, 2010; Thompson et al., 2017), the chain length of the steric stabilizer used was fixed. Here, four pMMA-b-pDMAEMA di-block copolymers with different DMAEMA block lengths (where the MMA block length was fixed at ~14–16 units and the DMAEMA content was varied from ~60 to 95 mol%) were investigated in terms of their free polymer behavior and the dispersion stability of synthesized core-shell latex particles. The ability of particles to stabilize emulsions and undergo rapid phase separation and release from pH changes are also characterized. Finally, these sterically stabilized latex particles are used to produce size-controlled low polydispersity droplets using membrane emulsification, for continuous scalable manufacture.

## Materials and methodology

### Materials

The chemicals used in this study are listed with details of purity and suppliers. Methyl methacrylate (MMA, purity ≥99%, Sigma Aldrich) and dimethylaminoethyl methacrylate (DMAEMA, ≥98%, Sigma Aldrich) monomers were purified by distillation prior to use. Cyanopropyl dithiobenzoate (CPDB) was synthesized and purified according to the protocol described elsewhere (Moad et al., 2000) and is used as a Reversible addition–fragmentation chain transfer (RAFT) agent. Azoisobutylnitrile (AIBN, >98%, Fluka) was purified by recrystallization from hot methanol prior to use. All other chemicals listed as follows were used as received: toluene (>99%, Fisher Scientific), dichloromethane (>99%, Acros Organics), hexane (>97%, Sigma), styrene (>99%, Sigma-Aldrich), ammonium persulfate (APS, >98%, Sigma-Aldrich).

### Synthesis of the responsive copolymer

The pMMA-b-pDMAEMA di-block copolymer used in this study was prepared, purified and characterized according to the protocol described by Cayre et al. (2012) (where DMAEMA_245_ was used). For this study, the MMA block length was fixed (14–16 units) and the DMAEMA length was altered to 20, 54, 108, and 245 units (equating to a DMAEMA content of ~60–95 mol% of the total polymer molecular weight) to compare their properties in solution, as a steric stabilizer and finally as an emulsifier. A summary of the four copolymers and their molecular weights used in this study are presented in Table 1.

**Table 1:** Summary of the copolymer block length, molecular weight, and polydispersity of the four pMMA-b-pDMAEMA copolymers used in this study.

**Stabilizer**	**Copolymer block length**	**Average molecular weight, M_n_ (g mol^−1^)**	**DMAEMA mol %**	**Polydispersity (PDI)**
DMAEMA_20_	pMMA_14_-b-pDMAEMA_20_	4,770	58	1.1
DMAEMA_54_	pMMA_14_-b-pDMAEMA_54_	10,110	79	1.2
DMAEMA_108_	pMMA_14_-b-pDMAEMA_108_	18,600	88	1.2
DMAEMA_245_	pMMA_16_-b-pDMAEMA_245_	40,340	94	1.1

### Characterization of solution properties of the di-block copolymer

#### Dynamic light scattering (DLS)

Measurements were made at 25°C using a Brookhaven BI-200SM instrument equipped with a 633 nm Helium-Neon (He-Ne) laser. To estimate the critical micelle concentration (CMC) of the di-block copolymers, changes in the hydrodynamic diameter were measured in polymer solutions prepared at different concentrations. Typically, a stock polymer solution was prepared at pH 4 at a concentration of 1,000 ppm. This was diluted with Milli-Q water also at pH 4, to obtain different polymer solution concentrations varying from 50 to 1,000 ppm (in this case there is no further dilution). The angle studied for the light scattering measurements was 90°. All copolymer solutions analyzed by DLS were passed through a syringe-mounted 0.2 μm filter.

#### Potentiometric titrations

All aqueous solutions were prepared by molecularly dissolving the copolymer in dilute HNO_3_ (pH 2; Milli-Q grade water), with constant background electrolyte of 0.01 M KNO_3_. Potentiometric titrations were performed by titrating 1,000 ppm copolymer solutions with 0.01M KOH from pH 2 to 11 (Figure S1 in Supplementary Information). Probe calibration was carried out using pH 4, 7, and 10 buffers.

### Synthesis and characterization of the responsive latex particles

#### Synthesis protocol

The responsive polystyrene latex particles were prepared via emulsion polymerization at different reaction temperatures, based on the method described in previous work (Cayre et al., 2012). Typically, the diblock copolymer stabilizer (0.5 g) was added to Milli-Q water (45 ml) (adjusted to pH 3–4 using HNO_3_) in a three necked 100 mL round bottom flask fitted with a reflux condenser and a magnetic stirrer. This mixture was stirred at room temperature to allow the stabilizer to dissolve, before placing the flask in an oil bath and heating it to a working temperature of 70°C. The reaction was purged with nitrogen and an aqueous solution of ammonium persulfate initiator (0.05 g) (1.0 wt% based on styrene) was added to the vessel. The styrene (5 ml) was then added and the polymerization was allowed to proceed for approximately 24 h. Serum replacement was used to remove excess stabilizer, trace monomer and initiator, and was performed by using dialysis tubing (Fisher, M_w_ = 12–14 kDa) over a time period of 1 week.

#### Dynamic light scattering (DLS) and zeta (ζ)-potential

Measurements were performed using the Zetasizer Nano ZS (Malvern, U.K.) equipped with a helium-neon laser with a wavelength of 633 nm. DLS was used to measure the changes in hydrodynamic diameter of sterically-stabilized latex particles as a function of pH, electrolyte concentration and temperature. The angle studied for the light scattering measurements was 173°. The influence of pH and electrolyte concentration on the zeta potential was also measured. The solution pH was adjusted by adding either HNO_3_ or KOH.

#### ^1^H NMR spectroscopy

The grafting density of the stabilizing diblock copolymer on the particles was determined through NMR studies of particles dissolved in deuterated chloroform. The intensity signals from the polystyrene and stabilizer were analyzed via integration of the relevant proton signals (Amalvy et al., 2004). It was assumed that the stabilizer was uniquely located on the surface of the particles. The grafting density, Γ, was calculated by comparing the stabilizer content (which takes into account the integration peaks) with the available particle surface area (based on intensity-averaged particle diameter using DLS).

### Preparation of batch emulsions via homogenization

For the bulk emulsions study, an IKA T25 Ultra-Turrax homogenizer operating at 15,000 rpm for 2 min was used. The aqueous phase was prepared at a 2 wt% latex dispersion into Milli-Q water (5 ml) and was adjusted using HNO_3_ or KOH to obtain the desired solution pH. Prior to emulsification, the latex dispersion was sonicated for 20 min before the emulsification experiments were carried out. An equal volume of n-hexadecane was added and the two phases were homogenized. The final emulsions were then placed in a water bath at 25°C for 24 h to assess their stability, and were characterized using laser diffraction and by monitoring the movement of the oil-emulsion and emulsion-water phases.

### Preparation of emulsions using rotational membrane emulsification

Membrane emulsification studies were conducted using a stainless steel membrane mounted on an overhead stirrer motor (IKA, Eurostar digital agitator) that was carefully positioned in a stationary cylindrical container. The steel membrane had an array of 108 square pores with a pore size of 80 μm × 80 μm used in a previous study with silica nanoparticles (Manga et al., 2012). The membrane rotational speed was kept constant in a given experiment; rotational speeds were systematically varied here from 500 to 1,500 rpm. The oil injection rate was controlled using a syringe pump (Razel A99FMZ, Fisher Scientific, UK) with a wide range of pumping rates from 0.075 × 10^−6^ to 75 × 10^−6^ m^3^ h^−1^ (corresponding to oil flow rates of 10^−3^ to 1 ml min^−1^) and a total oil volume of 5 mL was injected for each experiment. The continuous phase used in each experiment was 25 mL with particle concentrations of 2 and 4 wt%. The optimal pH and electrolyte concentration to prepare emulsions were based on the findings from the bulk emulsion studies prepared using the homogenizer. Prior to emulsification, the latex dispersion was sonicated for 20 min before the emulsification experiments were carried out.

These emulsions were characterized to obtain the number average droplet diameter measured from optical microscopy images. To minimize distortion of the droplets during analysis pipettes with a wide opening were used and were imaged without glass cover slides. Optical microscopy was used here, as some of the droplets produced were too large for size measurement using standard laser diffraction techniques (e.g., Malvern Mastersizer). The average diameter and standard deviation of the emulsions was determined by manually measuring several hundred droplets.

## Results and discussion

### Solution properties of the copolymer

The di-block copolymers used are comprised of a hydrophobic block (MMA) and a hydrophilic block (DMAEMA). The latter allows for the resulting polymer to be readily soluble in weakly acidic aqueous environments (pH ≈ 4) due to the homopolymer exhibiting a p*K*_a_ value between 7 and 7.5 (Baines et al., 1996). Indeed, the DMAEMA block contains a tertiary amine group that protonates as the pH decreases below the polymer p*K*_a_, thus dictating the block solubility in water as a function of pH. Similar block copolymers have been reported to form micelle structures in solvents, which are selective for one of the blocks (Tuzar and Kratochvíl, 1976; Price, 1982; Munk et al., 1992). The study and characterization of such micellar structures is important when considering their role in the synthesis of latex particles via emulsion polymerization (Lovell and El-Aasser, 1997).

**Figure 1:**
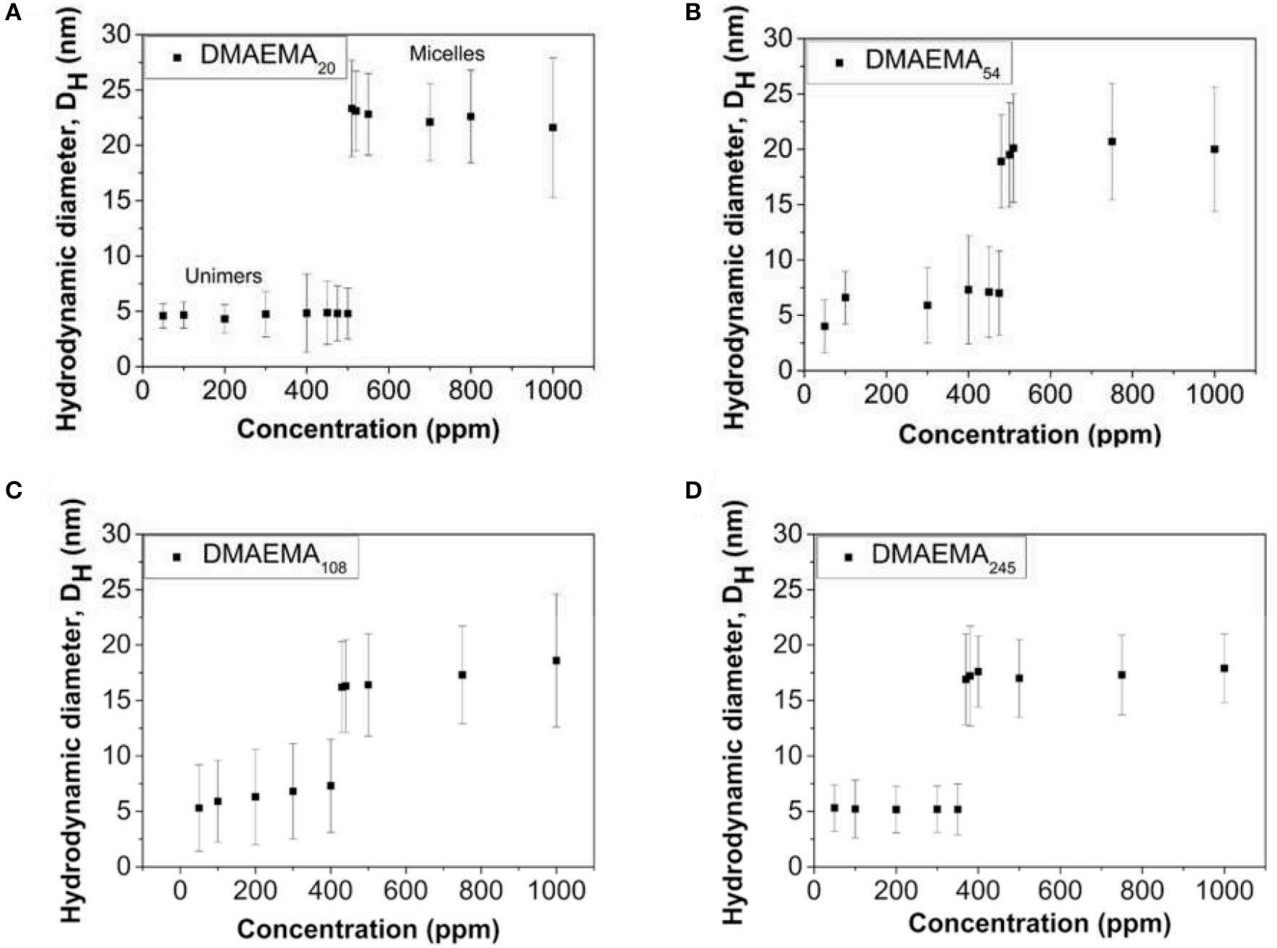
Variation in the diblock copolymer (or the formed micellar aggregates) hydrodynamic diameter as a function of polymer concentration for four different DMAEMA block lengths; **(A)** 20, **(B)** 54, **(C)** 108, and **(D)** 245. The polymer solutions are prepared at pH 4 in the presence of 0.01 M KNO_3_ (*T* = 25°C).

DLS measurements were performed to measure the changes in hydrodynamic diameter as a function of polymer concentration and DMAEMA block length, prepared at pH = 4 (below the pKa of the DMAEMA block, resulting in a protonated and thus water-soluble polymer) in the presence of 0.01 M KNO_3_ electrolyte. The resulting changes, for all four DMAEMA block lengths synthesized are shown in Figure 1.

Although information regarding the individual size of the micelle core and corona cannot be gathered using DLS (Brookhaven), the measured hydrodynamic diameter gives indication of the overall size of the copolymer aggregates in solution. The data in Figure 1 show that at low polymer concentrations (below 400–500 ppm) the copolymer exists as unimers, which results in smaller hydrodynamic diameters being measured. When the polymer concentrations reach a critical concentration and above, these individual chains self-assemble into micellar structures consisting of a hydrophobic core (MMA block) and a hydrophilic corona (DMAEMA block). The variation associated with the values presented in Figure 1 are large, but they do seem to suggest that as the DMAEMA block length increases, the overall micellar aggregate diameter at pH 4 (at the cmc value) appears to decrease. This apparent decrease in hydrodynamic size matches similar observations reported by Xiao et al. (2012) where the change can be described by the packing parameter theory (Eastoe, 2005). As the DMAEMA block length increases, it leads to a hydrophilic head group occupying a larger volume, driving a higher degree of curvature for the assemblies with a corresponding smaller aggregate size.

The transition from unimers to equilibrium micellar aggregates occurs at different values as the DMAEMA content changes. An increase in the block length leads to an overall decrease in the CMC (in accordance with the standard definition, and known as CMC). This relationship is due to the fact that as the DMAEMA block length increases, the overall volume occupied by the headgroup also increases leading to greater separation distances between neighboring hydrophobic chains within the core (reduced density of unimer chains participating in the micelle structure). Therefore the energy transition point (where micelles represent the lower energy state to minimize solvophobic interactions) is reached at lower concentrations (Karayianni and Pispas, 2016). The cmc values reported here using dynamic light scattering are of the same order that were reported by Baines et al. (1996), i.e., 0.5 g/L using surface tension measurements, where the diblock copolymer studied was comprised of ~80 mol% DMAEMA (similar to DMAEMA_54_ in our case).

**Figure 2:**
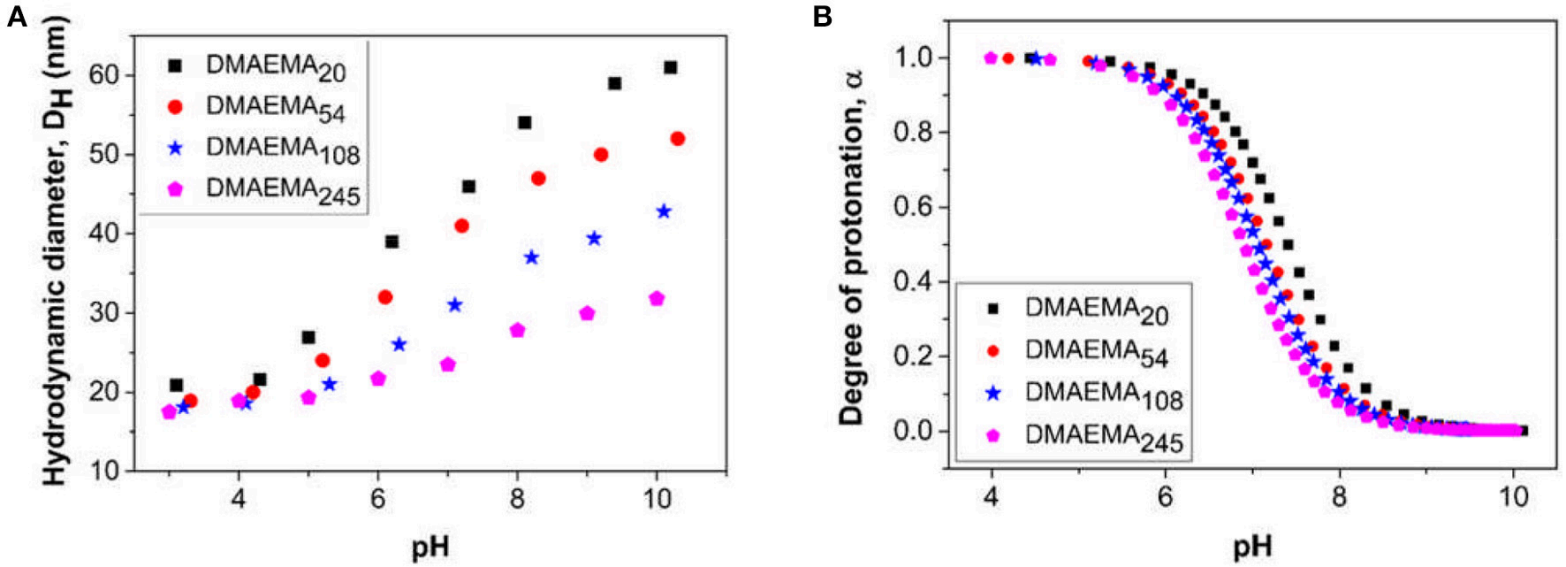
Changes in **(A)** hydrodynamic diameter and **(B)** degree of polymer protonation as a function of solution pH for the four diblock copolymers prepared at a concentration of 1,000 ppm (above the CMC). The polymer solutions are prepared in the presence of 0.01 M KNO_3_, (*T* = 25°C). The p*K*_a_of the copolymers obtained are; DMAEMA_20_ = 7.41, DMAEMA_54_ = 7.16, DMAEMA_108_ = 7.08, and DMAEMA_245_ = 6.92.

The changes in hydrodynamic diameter of the micellar aggregates (at 1,000 ppm i.e., above cmc at pH 4) as a function of solution pH was also measured to study the effect of DMAEMA block protonation/deprotonation (Figure 2A). The hydrodynamic diameter of the micellar aggregates increases as a function of increasing solution pH, which can be explained by examining the data from the pH titration of the polymer (Figure 2B). At low pH values (pH 4 and below) the amine groups are fully protonated leading to high surface charge densities. This protonation increases the electrostatic repulsive forces exhibited between neighboring chains and as a result the chain packing density will reduce to accommodate for the larger volume occupied by the hydrophilic block, leading to an evolution of the self-assembled objects toward smaller aggregates containing fewer polymer chains. As the pH is increased, the DMAEMA block becomes increasingly deprotonated (majority of the polymer dissociation occurs over a narrow pH range), resulting in weaker electrostatic repulsive forces, which allows for more efficient packing of the polymer chains leading to micellar growth (Wesley et al., 2005). These trends are confirmed by similar observations found by Xiao et al. (2012). The degree of protonation data confirms that the DMAEMA block is weakly basic with a p*K*_a_ value of around 7–7.5 (Amalvy et al., 2004), which slightly decreases with increasing pDMAEMA block length matching similar observations made in previous work (Van De Wetering et al., 1998). Similarly, increasing the DMAEMA monomer content at low pH values leads to the formation of seemingly smaller micellar aggregates, likely containing fewer polymer chains, due to the stronger repulsive forces resulting from the higher concentration of charges in the micelle coronas.

### Synthesis of sterically stabilized latex particles

#### Effect of DMAEMA block length

Emulsion polymerization of styrene in the presence of the di-block copolymers was performed to investigate the influence of the DMAEMA block length stabilizer on the size of the resulting latex particles and their bulk properties. To determine the effect on particle size only, measurements using DLS and Scanning Electron Microscopy were performed at pH 8. At this pH, it was assumed that the polymer would retract back onto the surface of the latex particles, but not lead to flocculation and therefore, allow comparisons of the particle core size using both techniques. Size measurements of the latex particles are presented in Table 2, scanning electron micrographs are presented in Supplementary Information (Figures S2.1, S2.2) where all particles synthesized were spherical in shape.

**Table 2:** Influence of DMAEMA block length on latex particle diameters synthesized with the various diblock copolymers used as stabilizers in the emulsion polymerization process.

**Sample**	**Number of DMAEMA units in the block copolymer**.	**DLS latex particle hydrodynamic diameter (nm) (polydispersity)**	**SEM latex particle (dry) diameter (nm) (polydispersity)**
LP-DMAEMA_20_	20	–	–
LP-DMAEMA_54_	54	57 (0.06)	53 (0.06)
LP-DMAEMA_108_	108	68 (0.08)	64 (0.09)
LP-DMAEMA_245_	245	87 (0.05)	84 (0.07)

*Mass of polymer stabilizers and reaction temperatures (70°C) were kept constant*.

The polymerization in the presence of the smallest diblock, i.e., DMAEMA_20_ (LP-DMAEMA_20_) led to an uncontrolled process that resulted in the formation of particles with high polydispersity on numerous occasions and was therefore not further evaluated as part of this study. This issue was not observed with the other three di-block copolymers. The data in Table 2 illustrates that the latex particle diameter generally increases as the pDMAEMA block length increases. For the polymerization reaction, the mass of polymeric stabilizers was fixed at 0.5 g which was well above the polymer CMC values (>10,000 ppm) outlined in Figure 1. However, it is worth noting that fixing the mass of polymer corresponded to a decrease in the molar concentration of the polymer in solution (i.e., number of polymer chains) with increasing DMAEMA content (by virtue of increasing molecular weight) which is summarized in Table S3 in the Supplementary Information. It was assumed the molar reduction may play a role in the reaction initiation process, as well as clearly particle growth and stability. Indeed, it is evident that the increase in latex particle size may be caused by the reduction in stabilizer units for the larger DMAEMA block copolymers, as the lower total number of chains would therefore not be able to stabilize the same total particle surface area as the smaller units.

#### Polymer grafting density

The grafting density of the polymer chains onto the latex particle provides important information about how the polymer stabilizer molecular weight may influence the properties of the resulting core-shell latex particles. To measure the polymer chain grafting density on the surface of the particles, ^1^H NMR spectroscopy experiments were performed after dissolution of the latex particles in CDCl_3_. The block copolymer grafting densities of all successful latex particles samples are presented in Table 3.

**Table 3:** Number of polymer chains and amine groups per particle derived from ^1^H NMR experiments carried out in CDCl_3_ after dissolution of the synthesized particles.

**Sample code**	**Molecular Weight**	**Adsorbed amount, Γ (mg.m^−2^)**	**Chains/nm^2^**	**Chains per particle**	**Amine groups per particle**
LP-DMAEMA_54_	10,110	0.23	0.018	53	2,862
LP-DMAEMA_108_	18,600	0.24	0.0072	42	4,536
LP-DMAEMA_245_	40,340	0.3	0.0045	29	7,105

The data in Table 3 show that the number of polymer chains occupied per unit surface area of the particle decreases with increasing DMAEMA block length. As discussed, because the mass of the stabilizer used in the polymerization process was fixed, increasing the DMAEMA meant that there are fewer chains in solution to begin with (due to higher polymer molecular weight) and a resultant formation of larger particles. Additionally, the fact that the grafting density also reduced, is also indicative of the greater steric repulsions of the larger DMAEMA head groups.

The grafting densities obtained by ^1^H NMR can be compared with theoretical values that one would expect (summarized in Table S3 in the Supplementary Information) assuming that (i) all the styrene monomer is converted into polystyrene particles, (ii) using mean particle size to calculate total particle surface area based on SEM measurements and (iii) all the polymer chains are located on the particle surface. These theoretical grafting densities also decrease with increasing DMAEMA content using particle sizes (based on mean diameters via SEM) obtained during polymerization. The theoretical grafting density values are around 20–30% higher than those calculated using ^1^H NMR, as in reality the polymerization conversion is less than 100%. The reduced number of polymer chains and grating density in turn affects the number of amine groups available per particle, which ultimately dictates the behavior of the particles in suspension and as emulsifiers in response to environmental pH and electrolyte conditions.

### Influence of pH and electrolyte concentration on the latex particle suspension properties

**Figure 3:**
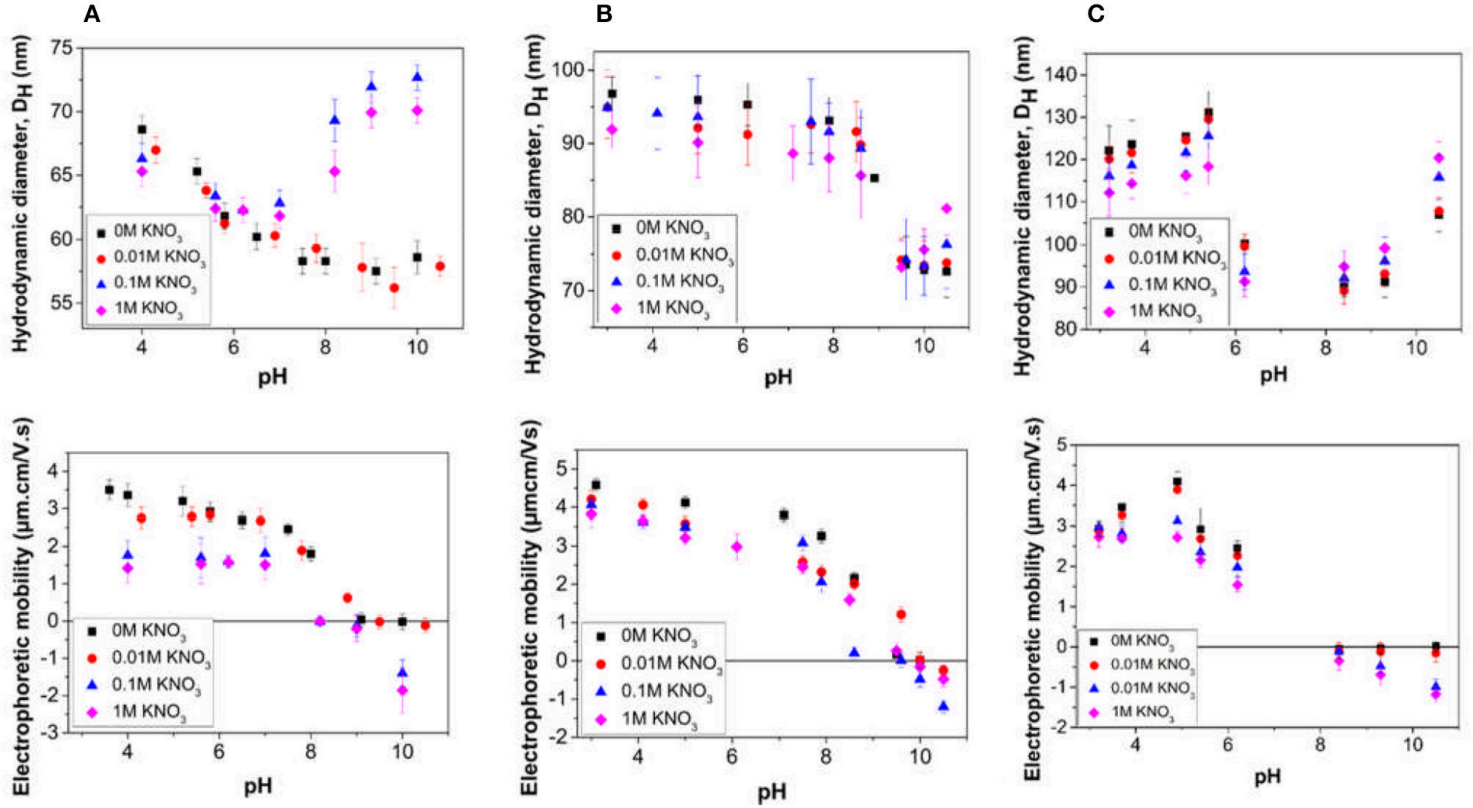
Influence of pH and electrolyte concentration on the properties of particle dispersions obtained from emulsion polymerizations conducted in the presence of the different diblock copolymers **(A)** LP-DMAEMA_54_, **(B)** LP-DMAEMA_108_ and **(C)** LP-DMAEMA_245_. Electrolyte concentrations used for the measurements were (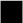) 0 M (i.e., no added) KNO_3_, (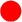) 0.01 M KNO_3_, (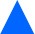) 0.11 M KNO_3_, and (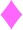) 1 M KNO_3_.

The effect of pH and electrolyte concentration on the properties of the particle dispersions was investigated by measuring changes to the hydrodynamic diameter and electrophoretic mobility, for the successful particle synthesis samples (LP-DMAEMA_54_ to LP-DMAEMA_245_) and are presented in Figure 3. The behavior of the samples in suspension is observed to be significantly dependent on the stabilizer chain length.

For sample LP-DMAEMA_54_ (Figure 3A), as the pH is decreased from around pH 8 to pH 4, there is a clear increase in the electrophoretic mobility toward a plateau value, as a result of the protonation of the amine groups on the DMAEMA. This behavior is consistent with the pure polymer protonation (Figure 2).The charging also results in an observed slight increase in the measured particle diameter, assumed to be due to extension of the grafted and charged polymer stabilizer chains. It is interesting that for this sample, at very low pH values, the mobility is relatively constant, whereas the diameter seems to further increase as the pH reduces. Although the number of polymer chains present per surface area is highest for this system (Table 3) the number of amine groups present per particle is the lowest of the three samples. Steric hindrance from the relatively high grafting density may influence the hydrodynamic response of the polymer at these low pH conditions and further work is needed to fully understand this behavior.

Increasing the pH of the LP-DMAEMA_54_ particle dispersions above 8 results in an increase to the measured particle size (especially at higher salt levels) whilst the measured mobility remains close to zero. At high pH, the polymer on the particle surface is deprotonated and as a result it is no longer soluble in the bulk, therefore collapsing onto the particle surface. The measured increase in the hydrodynamic diameter is most likely caused by the formation of particle aggregates driven by inter-segmental attraction between the uncharged DMAEMA chains on the surface of approaching particles. Such changes will be further driven through salt coagulation.

For the latex particles sterically stabilized by LP-DMAEMA_245_ (Figure 3C) there is a contrast in the particle hydrodynamic diameter changes below the polymer p*K*_a_, where the measured size reaches a maximum value at pH 5 before decreasing with lower solution pH. A potential reason for this behavior is that while the number of polymer chains per particle is lower than LP-DMAEMA_54_, the larger chain length occupies a larger volume per particle surface area, and overall, there are many more charge groups per particle (Table 3). These differences make it more susceptible to changes in pH and electrolyte conditions. Therefore, as the pH is decreased, it is more likely that there is a salting effect from the high electrolyte levels at low pH, which reduces the electrostatic repulsion between the chains and hence a reduction in overall size is observed. The effect of electrolyte is more evident for LP-DMAEMA_245_ at low pH, with measured diameters being lower for high salt conditions (attributed to greater salting-out effect). At high pH, there is also evident aggregation of the dispersions from destabilization of the particles as a result of the collapsed chains.

Aggregation of the particles appears to be less pronounced for the LP-DMAEMA_108_ sample (Figure 3B), which does not appear to be related to the amine moiety density (that varies with increasing block length). Instead, it is thought that this is a direct consequence of the surface charge distribution on the three different samples. Indeed, the electrophoretic measurements carried out for the LP-DMAEMA_108_ sample show clearly a shift to higher pHs for the point of zero charge of the particles. This is likely due to differences of initiator species incorporation on the particle surfaces between the different latex samples, as these species carry a negative charge, which can compensate for some of the positive charges resulting from the polymer. Nevertheless, the observed difference in the point of zero charge is likely to be responsible for the lack of aggregation of the LP-DMAEMA_108_ sample at the high pH values tested in this study, although it is expected that aggregation for this sample is likely to occur at slightly higher pHs.

### Influence of DMAEMA block length on pickering emulsion stability

**Figure 4:**
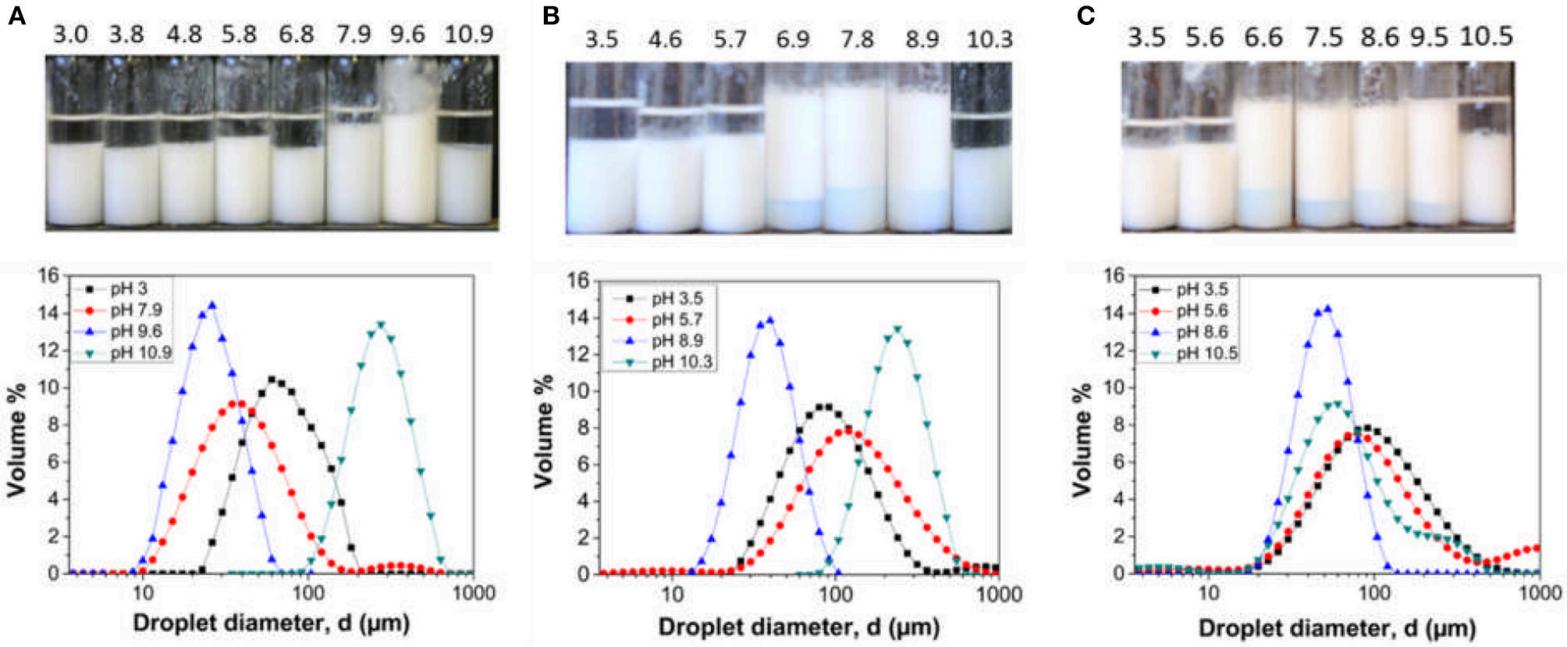
Hexadecane in water emulsions stabilized by **(A)** LP-DMAEMA_54_, **(B)** LP-DMAEMA_108_ and **(C)** LP-DMAEMA_245_ as a function of pH. Digital micrographs of the emulsions 24 h after production (top) and the droplet diameter distribution based on volume % (bottom). No background electrolyte was added to the continuous phase and measurements were performed a 25°C.

The three latex samples were investigated to determine their performance as Pickering stabilizers. Since the base particle size and the pMMA block lengths are similar, the influence of DMAEMA content on emulsion stability across the pH range can be assessed. Emulsions created at varying solution pH values with no added background electrolyte to the continuous phase are illustrated in Figure 4. The emulsions were left to stand at 25°C for 24 h prior to characterization.

The digital micrographs and droplet size data show varying degrees of emulsion stability as a function of solution pH. When examining emulsions created with LP-DMAEMA_54_, a highly stable emulsion with a mean droplet size of 25 μm is produced at pH 9.6, as the pDMAEMA chains become deprotonated at this pH and therefore collapse back on the surface of the particle (increasing the hydrophobic character of the particles) and thus improving particle wettability at the o/w interface. Increasing the pH to 10.9 leads to droplet coalescence (large polydisperse droplets) and macroscopic phase separation occurs. It is assumed this instability is due to adsorption of latex aggregates, driven by intersegmental attraction of the deprotonated pDMAEMA chains (as observed from increased dispersion size data in Figure 3) producing larger droplets that are destabilized. When the pH is decreased below 9.6 (pH 7.9–3), emulsions with macroscopic phase separation are observed. This change is due to the pDMAEMA chains becoming protonated, i.e., more cationic with increased solubility in the water phase, thus reducing their affinity for the interface. The observed behavior is in agreement with studies on similar responsive emulsion stabilizers reported previously (Amalvy et al., 2003, 2004).

When sample LP-DMAEMA_245_ is used as an emulsifier, the trend in emulsion stability/instability differs somewhat (as evidenced by differences in deprotonation pH from latex size and mobility in Figure 3). Although the same trend is found at pH 9.5 and above, decreasing the pH results in a wider band of pH for stable emulsions down to pH 6.6. Such changes may be explained by considering how the polymer chains behave as free polymers in solution vs. grafted onto a particle surface. In solution, the amine groups are highly susceptible to pH changes, and therefore the pH range for protonation is markedly narrow (as shown in Figure 2). When the same chains are grafted onto a particle surface, their response to environmental pH may be modified in magnitude or range. For larger chemically grafted polyelectrolytes, parts of the chain that are closer to the particle surface may remain unchanged, due to local screening effects leading to a distribution of charges along the polymer chain. Furthermore, at pH 9.5 where the polymer is deprotonated (i.e., in a collapsed state on the particle surface), decreasing the pH should begin to increase the degree of polymer protonation (based on the free polymer data presented in Figure 2). In reality, the protonation is most likely to be slower on a surface than when the polymer is dissolved in the bulk due to steric hindrance between the collapsed polymer chains. Lastly, the strength of the particle adsorption at the interface will be dictated by the polymer charge and its corresponding influence on the overall contact angle. Decreasing the pH below 6.6, the polymer degree of protonation increases and will drive a much larger affinity for the bulk for the particles, resulting in decreased particle adsorption energies and thus a more likely emulsion droplet coalescence with eventually macroscopic phase separation occurring.

The trend in emulsion stability when using LP-DMAEMA_108_ exhibits similar characteristics to LP-DMAEMA_54_ at low pH values and LP-DMAEMA_245_ around the polymer p*K*_a_ and above. The grafting density of LP-DMAEMA_108_ is similar to that of LP-DMAEMA_54_, but because of the larger polymer block length, the number of amine groups per particle is higher and thus their adsorption behavior as a function of pH lies in between that observed with LP-DMAEMA_54_ and LP-DMAEMA_245_.

**Figure 5:**
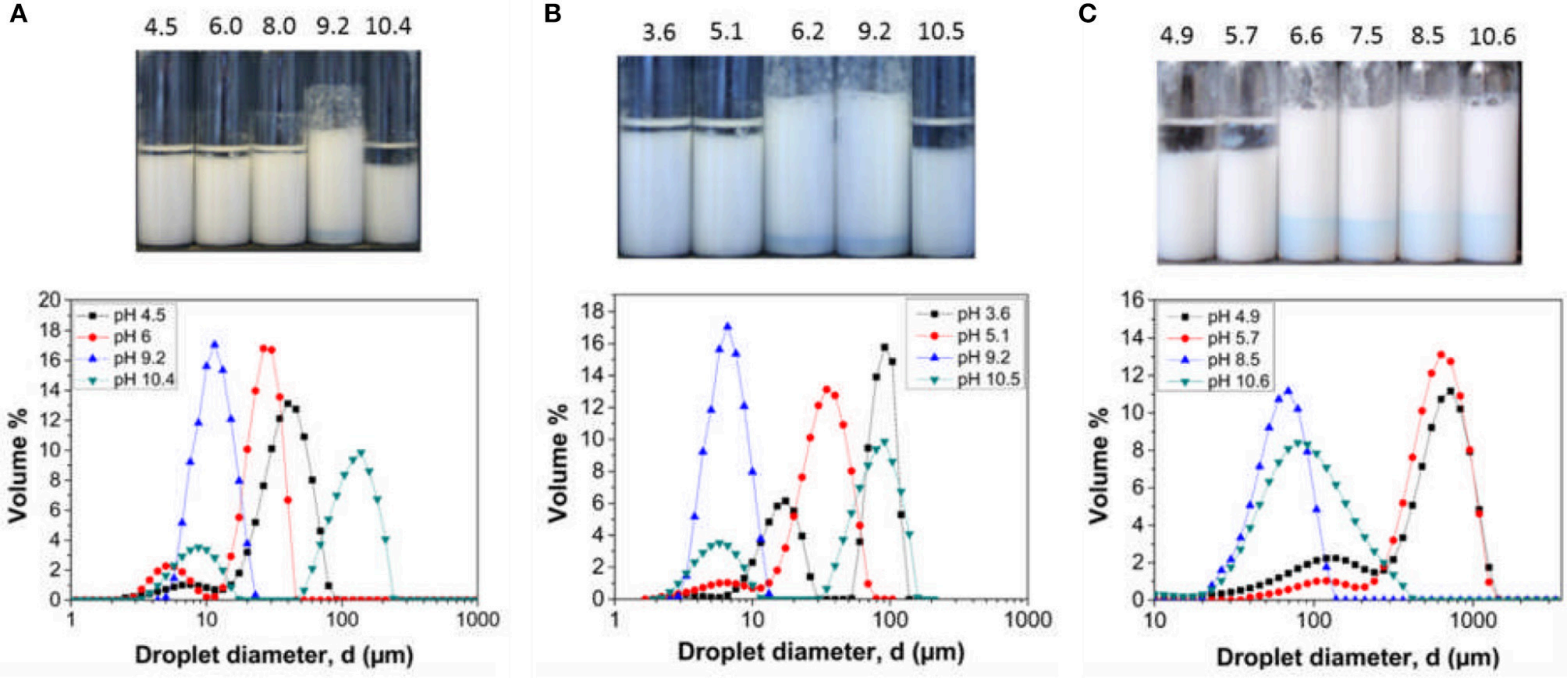
Digital images (top) and droplet size data (bottom) for hexadecane in water emulsions stabilized by **(A)** LP-DMAEMA_54_, and **(B)** LP-DMAEMA_108_, and **(C)** LP-DMAEMA_245_ as a function of pH in the presence of 0.01 M KNO_3_. Measurements were performed at 25°C.

The effect of adding background electrolyte on emulsion stability across the pH range was also examined. Digital micrographs and droplet size data of the emulsions created with 0.01 M KNO_3_ is presented in Figure 5.

The addition of background electrolyte to emulsions created with LP-DMAEMA_54_ shows an improvement in emulsion stability (volume of coalesced oil noticeably decreased) especially at the lower pH values. By adding electrolyte, screening of the protonated amine groups results in the polymer becoming less soluble in the continuous phase and it adopts a less extended configuration, enhancing the interfacial wettability of the latex particles. Additionally, low levels of background electrolyte (below levels causing bulk coagulation) may promote stronger interfacial films, with greater interfacial elasticity (Yu et al., 2017). This trend is confirmed by a shift in the droplet size data toward smaller sizes when compared to emulsion droplets created in the absence of background electrolyte. With LP-DMAEMA_245_, the effect of adding background electrolyte leads to improvements in emulsion stability at the extreme pH values examined. However, phase separation still occurs at pH 4.9 and 5.7, as the combination of adding background electrolyte and the electrolyte formed due to pH alteration (to reach these moderately acidic pH's) appears to not sufficiently screen the polymer charges in this case (as the number of charge groups is larger).

It is noted that, importantly, the emulsion droplet sizes (in the most stable pH region) are larger for the LP-DMAEMA_245_ samples than the particles with smaller charged block length. This difference is likely partially due to the particles being larger (as it is well known that smaller nanoparticles attain higher packing efficiencies and thus are better Pickering emulsifiers (Hunter et al., 2008). Differences may also be due to changes in particle wettability as the polymer length is altered, although, a previous study by Reed et al. (2012) on similar latex systems found that the particle contact angle at the interface was insensitive to the polymer chain length (PGMA_n_), as the polymer grafting density was high (larger than unity for all PGMA_*n*_–PS latexes studied). The grafting densities we obtain however are significantly lower, and as a result, the chain length may induce larger changes in particle wettability.

Despite the larger minimum droplet sizes, emulsions produced from LP-DMAEMA_245_ were considered more “responsive” than the other particles, as droplet sizes at lower pHs increased much more markedly (with mean sizes in the order of 1,000 μm). As a key potential application of such responsive Pickering systems would be the retention and release of oil-phase actives with a pH trigger, it was clear the LP-DMAEMA_245_ samples provided a better response envelope overall.

It is additionally noted, that increasing the concentration of the background electrolyte further resulted in larger emulsion droplets across the pH range investigated, although no macroscopic phase separation was observed (Figures S4.1, S4.2).

### Improving droplet size control via membrane emulsification techniques

Membrane emulsification is a technique to produce controlled droplets by expressing the dispersed phase through a porous membrane (with well-defined pores) in a drop by drop manner into a continuous phase containing the emulsifier. Droplet detachment occurs, due to shear forces acting on the membrane surface created by either a crossflow of the continuous phase over the membrane surface (Schröder et al., 1998; Williams et al., 1998; Bux et al., 2016), rotation/vibration of the membrane (Vladisavljević and Williams, 2006; Manga et al., 2012), or by mechanical stirring (Dragosavac et al., 2008; Thompson et al., 2011; Manga and York, 2017).

In this study, rotational membrane emulsification (RME) is used to explore the potential of using the sterically stabilized particles to produce controlled emulsions. The RME is a sensible rig to conduct such studies, as it allows for small scale experiments to be performed easily without the need for substantial quantities of the latex emulsifier. Based on the bulk emulsion studies, it was decided to perform the experiments at a solution pH of 9 and at a background electrolyte of 0.01 M KNO_3_ to ensure successful particle adsorption and stable emulsions (see Figure 5).

**Figure 6:**
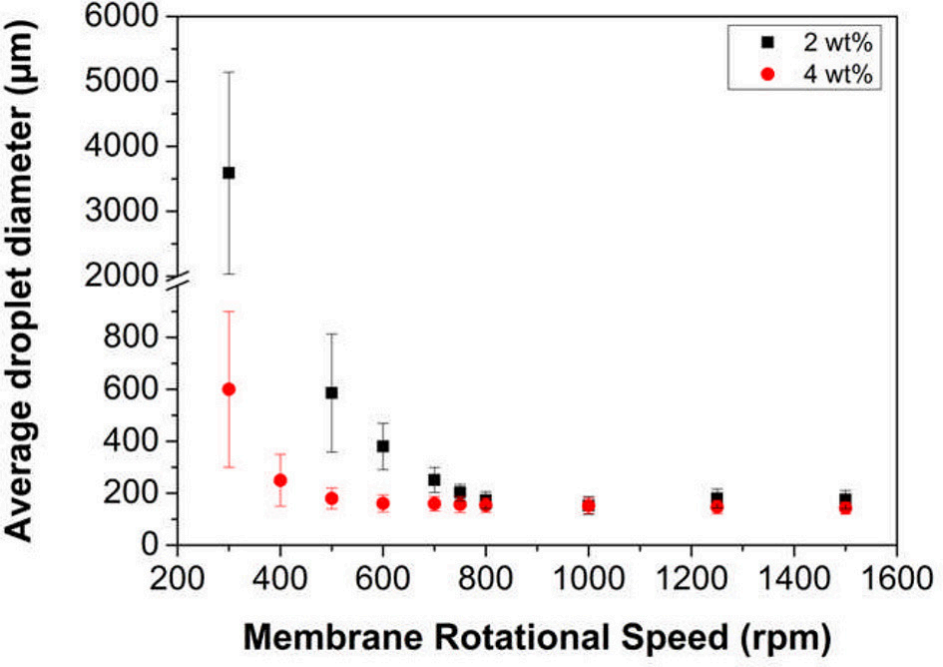
Variation in mean droplet size of hexadecane in water emulsions as a function of membrane rotation speed at 2 and 4 wt% particle loading of LP-DMAEMA_245_. The continuous phase contained an electrolyte concentration of 0.01 M KNO_3_ at pH 9. The oil injection rate was fixed at 0.01 mL min^−1^.

The residence time of the droplet at the membrane surface prior to detachment plays an important role in controlling the droplet size and size distribution of the emulsion (Manga et al., 2012). The residence time is controlled by the shear rate imparted at the membrane surface and in the case of RME this is done by the rotation of the membrane within the continuous phase. The influence of this rotation speed on droplet size and size distribution data when using LP-DMAEMA_245_ latex particles is presented in Figure 6. The oil injection rate was fixed at 0.01 mL min^−1^, which was selected based on the optimized conditions found previously when studying with 800 nm silica colloids (Manga et al., 2012).

In the case where a 2 wt% concentration of the latex emulsifier is used, large polydisperse droplets form at the lowest membrane rotation speed (i.e., 300 rpm). During the emulsification process at this speed, it is difficult to determine if these large droplets are a result of coalescence occurring in the bulk due to the opacity of the latex dispersion. The solution conditions as well as fast diffusion times of the particles from the bulk to the interface (due to their hydrodynamic diameter) should result in a stable emulsion with small diameters. However, the shear rate at the membrane surface is low and thus the droplets either (a) grow to a large size before detaching or (b) merge together from adjacent pores. As the speed is increased, both the droplet size and size distribution decreases with an apparent minimum occurring around 1,000 rpm. The increase in shear rate and fast particle adsorption times allow for such controlled droplets to be produced. It should be noted that there is very little difference in the droplet size for samples created between 800 and 1,500 rpm, representing the minimum size distributions possible from the membrane pore size.

Increasing the particle emulsifier concentration to 4 wt%, effectively increases the collision frequency between the particles and the interface from the greater particle loading, and resulted in improvements to both size and size distribution at the lower rotation speeds (300–600 rpm). This difference is driven by two significant factors. It is known that the grafted pDMAEMA is surface active, and will reduce the interfacial tension to equilibrium values much faster at higher particle loading (Manga et al., 2016) (higher collision rate with the interface). Additionally, the greater particle number will increase the total surface area that is able to be stabilized for a given particle loading, which together with surface tension reduction leads to the production of smaller droplets evident at the lower rotation speeds. However, at greater speeds, the size is similar for both particle concentrations, as the droplet breakup tends toward a minimum size value for a given oil flow rate, regardless of shear rate (over a certain threshold) dependent on the membrane pore size. This behavior is analogous to surfactant systems that have been studied (Vladisavljević and Williams, 2006) and matches a similar trend that was observed when 800 nm silica colloids were used (Manga et al., 2012). However, improvements in size and polydispersity are more pronounced with the latex particles, due to the influence of faster adsorption kinetics (from the smaller particle size) and surface active nature of the particles (Manga et al., 2016).

**Figure 7:**
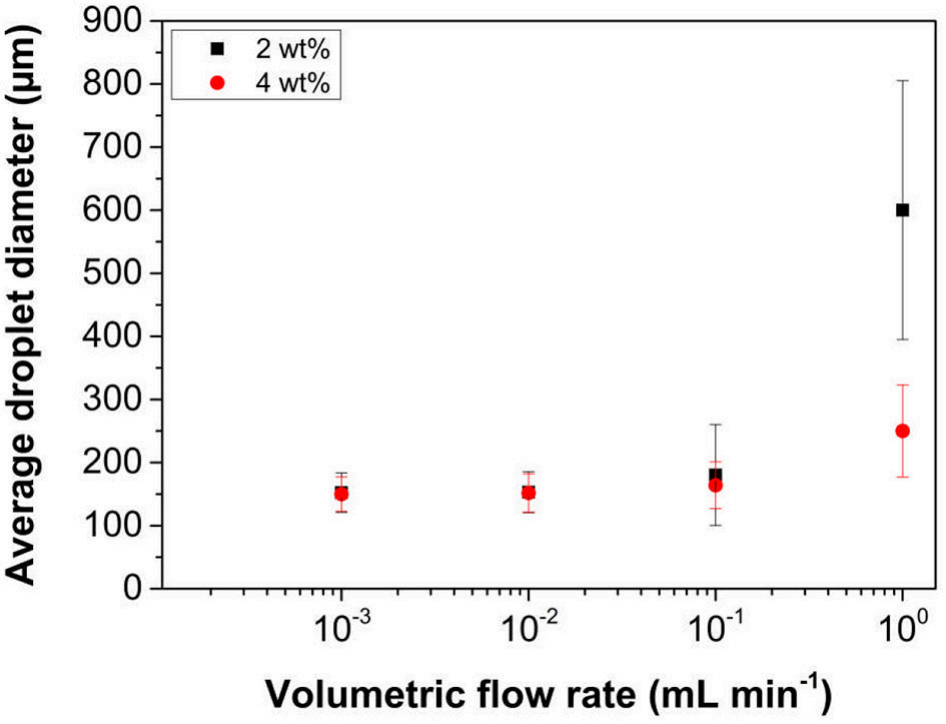
Variation in mean droplet size of hexadecane in water emulsions as a function of oil injection rate at 2 and 4 wt% particle loading of LP-DMAEMA_245_. The continuous phase contained an electrolyte concentration of 0.01 M KNO_3_ at pH 9. The membrane rotation speed was fixed at 1,000 rpm.

The oil injection rate through the porous membrane governs the rate at which new interfacial area is created, and therefore plays a critical role in controlling the droplet size and size distribution obtained. Additionally, from a production scale-up perspective, it is imperative to understand what are the maximum stable emulsion production rates possible for a given membrane size. Changes in mean droplet size with oil injection rate are presented in Figure 7, with a fixed membrane rotation speed of 1,000 rpm, again at two particle loadings.

At the fastest injection rate studied (1 mL min^−1^), emulsions with large polydisperse droplets are produced when using a latex concentration of 2 wt%. Increasing the concentration to 4 wt% dramatically causes a reduction in both the observed mean droplet diameter and associated size distribution. This reduction highlights the importance of adsorption kinetics, as by increasing the particle concentration the particle collision rate with the newly forming interface also increases. Decreasing the oil injection rate reduces the droplet sizes further to ~150 μm at 0.01 mL min^−1^. Since the shear force acting at the membrane surface is fixed, growth and detachment of the droplets from the membrane pore will occur at a fixed timescale. Therefore, size invariance at low injection rates, will most likely be due to high particle effective collision frequency and reduction of the interfacial tension, as the interface production rates are reduced below a certain critical threshold to produce stable droplets.

These data illustrate that the sterically stabilized latex particles can act as very efficient emulsifiers when using membrane emulsification technologies, provided the emulsification parameters have been fully optimized. With this single membrane device, for a production rate of 1 mL min^−1^ (where droplet coefficient of variation is 30% at a particle loading of 4 wt%) a 100 mL of emulsion (with a volume fraction of 20%) can be produced in 20 min. In comparison, microfluidic devices such as T-junctions are able to generate highly monodisperse droplets (size variation as low as 3%) however the flow rate of the disperse flow rate is very low, typically 0.01–10 mL h^−1^ (Basile and Charcosset, 2015). Taking the fastest rate, the example emulsion mentioned above would take almost 120 min to produce. The productivity can be increased by parallelization of the fluidic channels, however issues arise with regards to pressure drops, channel blockages and controlling the flow rates of individual streams in long channel networks. This is less of an issue in membrane emulsification, so by increasing the active membrane surface area or by parallelization, the overall throughput can be further increased.

## Conclusions

This study has demonstrated that DMAEMA monomer content plays a critical role in the synthesis of latex particles sterically stabilized by pMMA-b-pDMAEMA diblock copolymers, and their resulting performance as pH responsive Pickering emulsifiers. Increasing the pDMAEMA block length alters the solution behavior of these polymers, leading to a reduction in the CMC, which is an important parameter in latex synthesis via emulsion polymerization, as well as their hydrodynamic diameters and p*K*_a_. During synthesis of the latex emulsifiers, increasing the DMAEMA content of the steric stabilizer leads to formation of larger particles, due to the influence of monomer content on micelle numbers when a fixed polymer concentration by weight is used.

When used as emulsifiers, pH-responsive emulsions are obtained, which were typically stable above the polymer p*K*_a_, where macroscopic phase separation occurred below it, caused by the reduction in contact angle from particle charging. Destabilization was also evident at very high pH, and was assumed to be driven by particle aggregation in the bulk. The transition between emulsion stability and instability in the presence of very low electrolyte concentrations was dictated by the DMAEMA monomer content and its grafting density onto the latex emulsifiers. Overall, the largest block length stabilizer, resulted in the most pH responsive emulsions. Finally, it was shown that emulsions with well controlled sizes can be produced using a RME, providing the emulsification parameters are well optimized. In particular, the importance of the interplay between particle concentration vs. shear rate and rate of interfacial area production is presented.

## Author contributions

MM was primarily responsible for the collection of all data reported here and its initial analysis as well as aspects of the experimental design. MM was also the primary author of the article and was responsible for various drafts and final approval. OC, SB, and TH contributed to the analysis and interpretation of the data along with critical revisions to the draft manuscript. OC, SB, and TH also gave approval to the final version of the paper.

### Conflict of interest statement

The authors declare that the research was conducted in the absence of any commercial or financial relationships that could be construed as a potential conflict of interest.
