# Supplementary material for: Influence of pH-Responsive Monomer Content on the Behavior of Di-Block Copolymers in Solution and as Stabilizers of Pickering Latex Particle Emulsifiers
Source: Front Chem. 2018 Jul 20;6:301. doi: 10.3389/fchem.2018.00301 (PMC6062645; doi:10.3389/fchem.2018.00301)
Supplement: Supplementary file 1 [file Data_Sheet_1.docx]

Supplementary Material

Influence of pH-responsive monomer content on the behavior of di-block copolymers in solution and as stabilizers of Pickering latex particle emulsifiers

**Mohamed S. Manga,*^1^ Oliver J. Cayre,^1^ Simon Biggs,^1,2^ Timothy N. Hunter^1^**

*** Correspondence:** Corresponding Author: M.S.Manga@leeds.ac.uk

^1^ School of Chemical and Process Engineering, Faculty of Engineering, University of Leeds, Woodhouse Lane, Leeds, LS2 9JT, U.K.

^2^ The University of Western Australia, Crawley, Perth, Western Australia 6009, Australia

# S1. Potentiometric titration

A typical titration plot for a 1000 ppm polymer solution of DMA_20_ is presented in Figure S1.


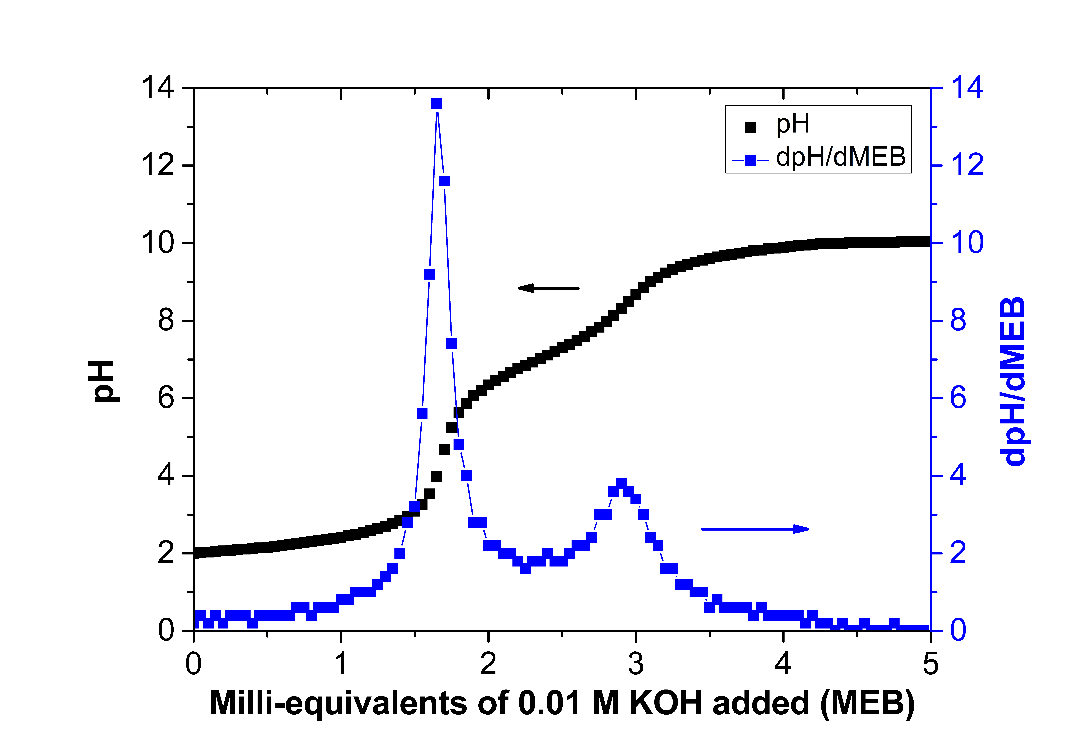


Figure S1. Potentiometric titration data typically obtained for the pMMA-b-pDMAEMA diblock copolymers. Plot of Δ*pH*/Δ*Volume of added base* illustrates the inflection points due to onset and complete proton association. Example given is for DMAEMA_20_ at polymer concentration of 1000 ppm.

In general, for a weakly basic polymer such as pDMAEMA, the titration curve will exhibit two inflection points, relating to the onset of proton association (inflection point 2) with the polymer and to complete proton association (inflection point 1), respectively. The positions of these inflection points are usually determined by plotting Δ*pH*/Δ*Volume of added base*. In all the studies considered here, the potentiometric titrations were conducted by titrating a base solution against an acidic copolymer solution. For every addition of KOH, the addition of hydroxide ions may undergo two possible interactions; either a) associating with and neutralizing a free proton in solution or b) neutralizing and associating with a proton from the tertiary amine site of the di-block copolymer. Another alternative is that the hydroxide ion does not undergo any of these interactions and instead remains as a free hydroxide ion within the solution.

# S2. Scanning Electron Micrographs of latex particles

**Influence of DMAEMA monomer content on particle size**


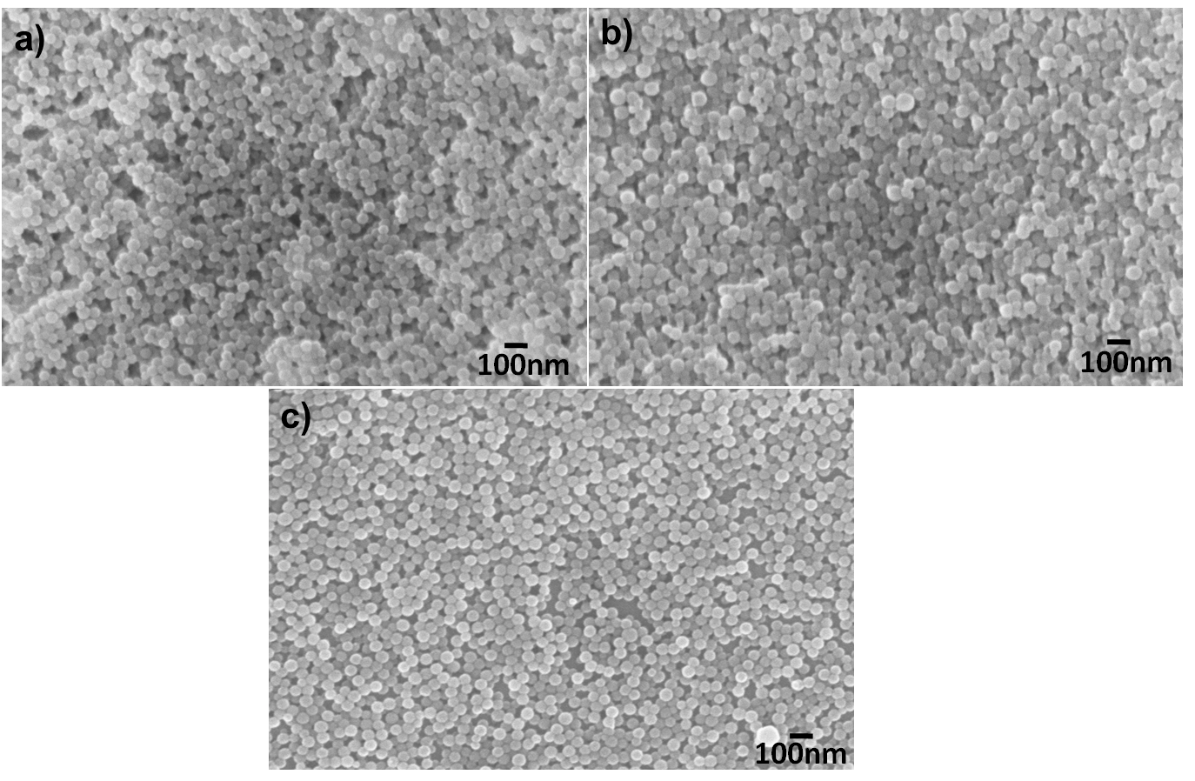


Figure S2.1. Scanning electron micrographs of polystyrene latex particles stabilized by varying pDMAEMA block lengths; a) 54 (LP-DMAEMA_54_), b) 108 (LP-DMAEMA_108_) and c) 245 (LP-DMAEMA_245_), produced via emulsion polymerization at 70°C.

Effect of reaction temperature on latex size at a fixed DMAEMA monomer content.


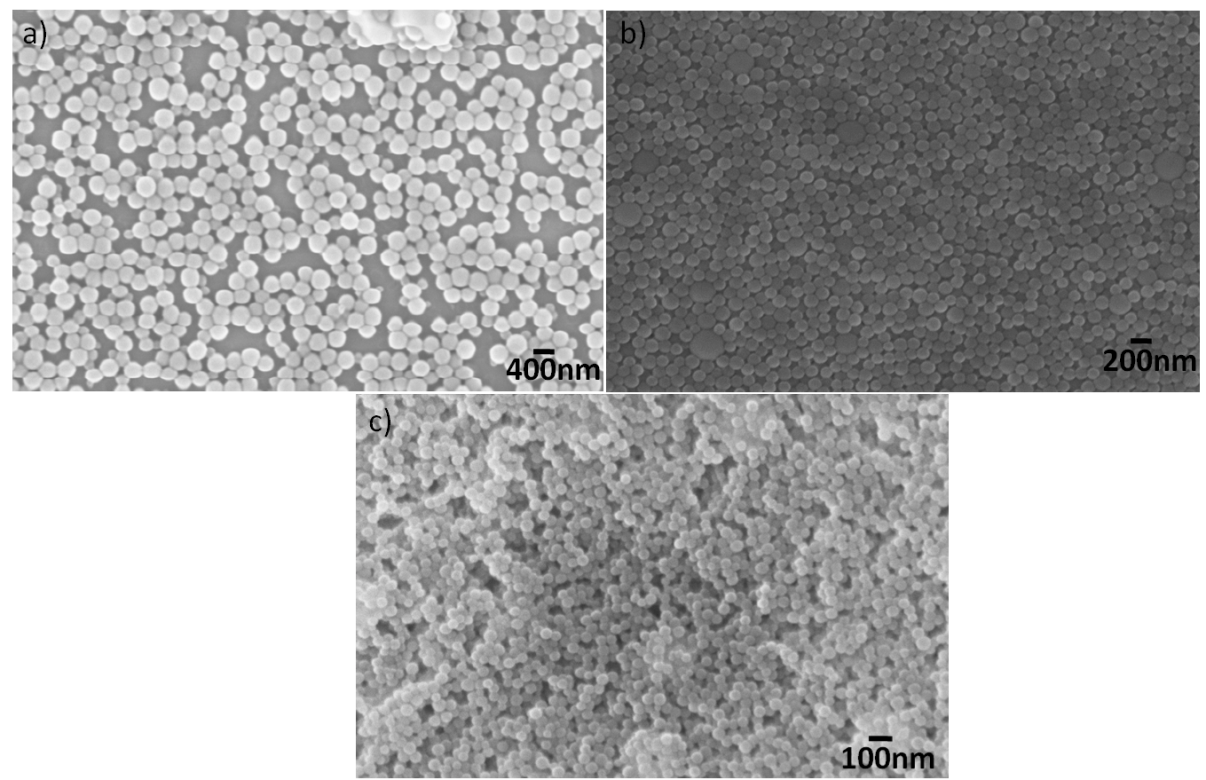


Figure S2.2. Scanning electron micrographs of polystyrene latex particles stabilized by pMMA_14_-b-pDMAEMA_54_, via emulsion polymerization at different reaction temperatures; a) 50°C, b) 60°C and c) 70°C (LP-DMAEMA_54_).

# S3. Grafting density measurements

**Table S3. Comparing polymer grafting density obtained from theoretical calculations vs. ^1^H NMR.**

| Sample | Molecular weight | Number of polymer chains (mol^-1^) | Surface area of particle (nm^2^) | Theoretical number of chains/nm^2^ | Actual number of chains/nm^2^ |
| --- | --- | --- | --- | --- | --- |
| LP-DMAEMA_54_ | 10110 | 2.97824E+19 | 8825.878 | 0.053 | 0.018 |
| LP-DMAEMA_108_ | 18600 | 1.61882E+19 | 12869.632 | 0.035 | 0.0072 |
| LP-DMAEMA_245_ | 40340 | 7.46406E+18 | 22169.952 | 0.021 | 0.0045 |

# S4. Characterization of emulsions created at 100 mM and 1 M KNO_3_ using both emulsifier systems.


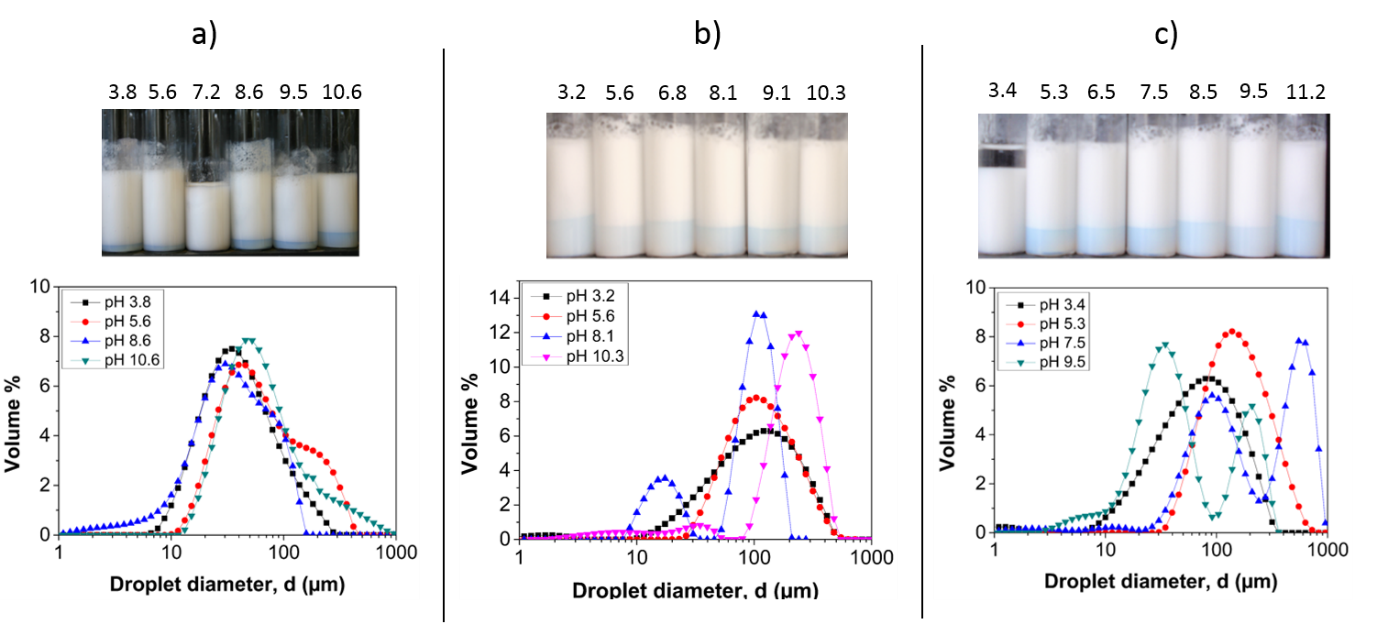


Figure S4.1. Digital images and droplet size data for hexadecane in water emulsions stabilized by a) LP-DMAEMA_54_ and b) LP-DMAEMA_108_ and c) LP-DMAEMA_245_ across the pH range in the presence of 0.1 M KNO_3_. Measurements were performed at 25ºC.


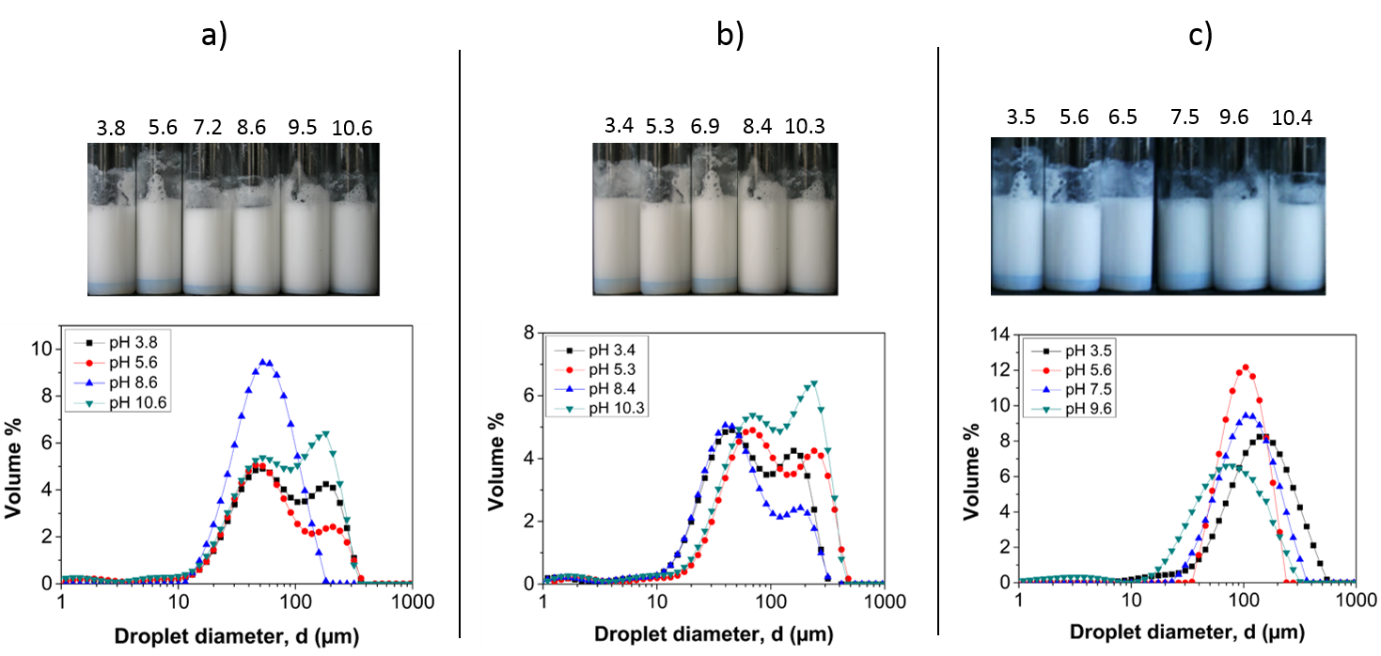


Figure S4.2. Digital images and droplet size data for hexadecane in water emulsions stabilized by a) LP-DMAEMA_54_ and b) LP-DMAEMA_108_ and c) LP-DMAEMA_245_ across the pH range in the presence of 1 M KNO_3_. Measurements were performed at 25ºC.
